# The effectiveness of exposure and response prevention combined with pharmacotherapy for obsessive-compulsive disorder: A systematic review and meta-analysis

**DOI:** 10.3389/fpsyt.2022.973838

**Published:** 2022-09-15

**Authors:** Lingyun Mao, Maorong Hu, Lan Luo, Yunhong Wu, Zihang Lu, Jingzhi Zou

**Affiliations:** ^1^Key Laboratory of Psychology of TCM and Brain Science, Jiangxi Administration of Traditional Chinese Medicine, Jiangxi University of Traditional Chinese Medicine, Nanchang, China; ^2^Department of Psychosomatic Medicine, The First Affiliated Hospital of Nanchang University, Nanchang, China

**Keywords:** exposure response prevention, obsessive-compulsive disorder, D-cycloserine, selective serotonin reuptake inhibitors, SSRIs, drug treatment, medication, meta-analysis

## Abstract

**Objective:**

To systematically evaluate the effectiveness of exposure and response prevention (ERP) combined with medication on obsessive-compulsive disorder (OCD).

**Methods:**

PubMed, Web of Science, EBSCO, Cochrane, Embase, and Science Direct databases were searched to include randomized controlled trials of ERP combined with medication for OCD that met the criteria. The Yale Brown Obsessive Compulsive Scale was used as the primary outcome indicator, and Depression scales were used as secondary outcome indicators. An evaluation of bias risk was conducted to identify possible sources of bias based on methodological and clinical factors. Review Manager 5.3 and Stata 16.0 software was used to perform meta-analysis of the extracted data.

**Results:**

A total of 21 studies with 1113 patients were included. Meta-analysis showed that ERP combined with medication therapy was significantly better than medication therapy alone including selective serotonin reuptake inhibitors, clomipramine and risperidone (MD = –6.60, 95% CI: –8.35 to –4.84, *P* < 0.00001), but D-cycloserine (DCS) drugs do not enhance the effect of ERP intervention in patients with OCD (MD = 0.15, 95% CI: –0.87 to 1.17, *P* = 0.77). There is more significant maintenance by combined treatment method of medication plus ERP than medication treatment alone during the follow-up period (MD = –7.14, 95% CI: –9.17 to –5.10, *P* < 0.00001). DCS drugs did not enhance the effect of ERP intervention on depression in patients with OCD (SMD = –0.08, 95% CI: –0.31 to 0.15, *P* = 0.50). ERP combined with drug improved patients’ depression levels significantly better than providing drug alone (SMD = –0.40, 95% CI: –0.68 to –0.11, *P* = 0.006).

**Conclusion:**

Patients with OCD have significant improvement in symptoms of obsessive-compulsive disorder and depression when ERP is combined with medication, however, not enough to prove that DCS can enhance ERP effectiveness.

## Introduction

Obsessive-compulsive disorder (OCD) is a psychological disorder characterized by recurrent intrusive thoughts and repetitive behaviors, which is frequently accompanied by anxiety or depression and has a lifetime prevalence of 2–4% (1). OCD is commonly detected at a late stage, and the longer untreated obsessive-compulsive symptoms persist, the less effective treatment and relapse prevention become (2). Therefore, finding an effective treatment and receiving appropriate treatment in a timely manner is crucial for OCD patients. Selective serotonin reuptake inhibitors (SSRIs) including fluoxetine, paroxetine, sertraline, and fluvoxamine are the first-line pharmacological treatments for OCD (3). However, only 40–60% of patients treated with SSRIs experienced a reduction in their obsessive-compulsive symptoms (4). Although tricyclic drugs (e.g., Chlorpromazine, Clomipramine, etc.) have better efficacy than SSRIs, their side effects are more pronounced and less endurable (5). Exposure and response prevention (ERP) is the first-line psychotherapy for OCD, and research indicates ERP-based therapy has comparable efficacy to SSRIs (4). Notwithstanding, the efficacy of ERP for OCD is highly variable, with approximately 25% of patients dropping out, 60% of patients recovering, and 25% of patients being successfully treated and cured (6).

In the clinical treatment of OCD, choosing medication or psychotherapy based on time and financial costs is a decision that patients need to consider. In order to achieve the best treatment outcome, it is more effective to combine psychotherapy and medication. For patients with mild or moderate symptoms, cognitive behavioral therapy (CBT) including ERP or SSRIs can be used alone, whereas CBT combined with SSRIs is recommended for patients with more severe or resistant conditions (7, 8). CBT combined with medication has shown better interventions in the clinical treatment of OCD, with all combined effect sizes at moderate to high levels (9). However, it has also been found that the efficacy achieved with the combination of sertraline and CBT was not sustained long-term, and that at 52 weeks, sertraline monotherapy was the most effective and cost-effective treatment (10). D-cycloserine (DCS) is a partial N-methyl-D-aspartic acid agonist that was initially utilized to treat tuberculosis (11). Researchers began to wonder whether DCS enhances exposure-based CBT based on the positive effects observed in animal studies investigating the fear-elimination paradigm. However, results were mixed (11). It is unclear whether DCS, as a novel clinical trial drug, will enhance the effectiveness of ERP treatment for patients with OCD.

Current studies have examined CBT combination pharmacotherapy for OCD (4, 12, 13). However, these studies have actually evaluated the effects of mixed forms of CBT, including cognitive therapy, behavioral therapy, and the combination of ERP and cognitive therapy. The study by Ost et al. (13) included 7 cognitive treatments with no components of ERP and 13 combinations of cognitive therapy and ERP. Nevertheless, ERP and cognitive therapy are essentially different theoretical underpinnings (7). Some variants of ERP include informal cognitive techniques (for instance, cognitive restructuring during exposures) and some forms of cognitive therapy include informal exposures (e.g., behavioral experiments that test the validity of the patient’s obsessional beliefs), blurring the distinction between these treatments (14). Cognitive therapy focuses on correcting dysfunctional beliefs about the existence or significance of intrusive thoughts, and its behavioral experiments were used to identify and modify patients’ irrational beliefs, rather than repeatedly confronting anxiety-provoking situations, as in ERP (15). As previous Meta-analyses have primarily focused on any form of CBT combination therapy rather than specifically ERP, their final effect sizes may be attributable to therapy overlaps. Moreover, major depressive disorder is the most common comorbid diagnosis of OCD (16). In this study, a Meta-analysis was therefore conducted on the intervention effects of ERP combined with pharmacological treatment on obsessive-compulsive and depressive symptoms in patients with OCD, providing a foundation for future clinical application.

## Methods

### Search

PubMed, Web of Science, EBSCO, Cochrane, Embase, and Science Direct were searched from build to May 1, 2022. The search term 1 was (exposure response prevention OR exposure and ritual prevention OR ERP OR EXRP) AND (obsessive-compulsive disorder OR obsessive compulsive disorder OR OCD). Search term 2 was (exposure OR ERP OR EXRP) AND (obsessive-compulsive disorder OR obsessive compulsive disorder OR OCD) AND (Randomized OR RCT OR RCTs OR random).

### Inclusion and exclusion criteria

Inclusion, screening, and exclusion criteria for the articles were developed in accordance with the PRISMA statement and PICOS principles. Inclusion criteria: (i) Study design (S): randomized controlled trials (RCT) (ii) Study Population (P): studies involving patients with comorbid disorders were considered as long as the primary diagnosis of OCD was present. (iii) Interventions (I): the experimental group received ERP combined with pharmacological intervention. (iv) Comparison (C): the control group received ERP or pharmacological intervention alone. (v) Outcome indicators (O): The Yale Brown Obsessive Compulsive Scale (Y-BOCS) score was used as the primary outcome indicator, and the secondary outcome indicators included Depression scales.

Exclusion criteria: (i) duplicate publications, (ii) not empirical research, (iii) not ERP interventions, (iv) no control groups, (v) not OCD, (vi) review article, (vii) conference abstract, (viii) not RCT, (ix) missing data, (x) not ERP combined with pharmacological intervention, (xi) not Y-BOCS, and (xii) reused data.

### Risk of bias and data extraction

The risk of bias evaluation of final included studies was conducted by two psychology master students according to the Cochrane Handbook version 5.0.1. The assessment included following 7 aspects: (i) random sequence generation; (ii) allocation protocol concealment; (iii) blinding of participants and trial personnel; (iv) blinding of outcome assessment; (v) completeness of outcome data; (vi) selective reporting of results; (vii) other biases. The evaluators judged the above 7 aspects as “low bias,” “high bias,” and “unclear,” respectively, and asked a third psychology master student to discuss the evaluation when there was a dispute.

Data were extracted from the final included articles, mainly including (i) the first author, year of publication, and nationality of the articles, (ii) the sample size, gender, and age of the experimental and control groups, (iii) the interventions, intervention duration and follow-up time of the experimental and control groups, and (iv) Mean (M) and Standard Deviations (SD) used for the outcome indicators.

### Statistical analysis

Review Manager 5.3 and Stata 16.0 software was used to conduct Meta-analysis on the extracted data. Firstly, the size of heterogeneity among the studies was judged. In Meta-analysis, the fixed-effects model assumes that the studies are homogeneous, while the random-effects model assumes heterogeneity between studies (17). *I*^2^-statistic reflects the proportion of the heterogeneity component in the total variance of the effect size, and *I*^2^-statistic > 50% indicates relatively substantial heterogeneity (18). If *P* ≥ 0.1 and *I*^2^-statistic < 50%, the fixed-effect model was used; if *P* < 0.1 and *I*^2^-statistic ≥ 50%, the random-effect model was used; when the heterogeneity was more obvious, Sensitivity analysis, Meta-regression analyses and Subgroup analysis was conducted to explore the source of heterogeneity. Statistical analyses were performed using Mean Different (MD) if the outcome indicators were measured with the same instrument, and Standardized Mean Different (SMD) if the measurement instruments were different. The outcome indicators were statistically analyzed using 95% Confidence Interval (95% CI) and *P* < 0.05 indicates that the difference is statistically significant.

## Results

### Article screening process and results

**FIGURE 1:**
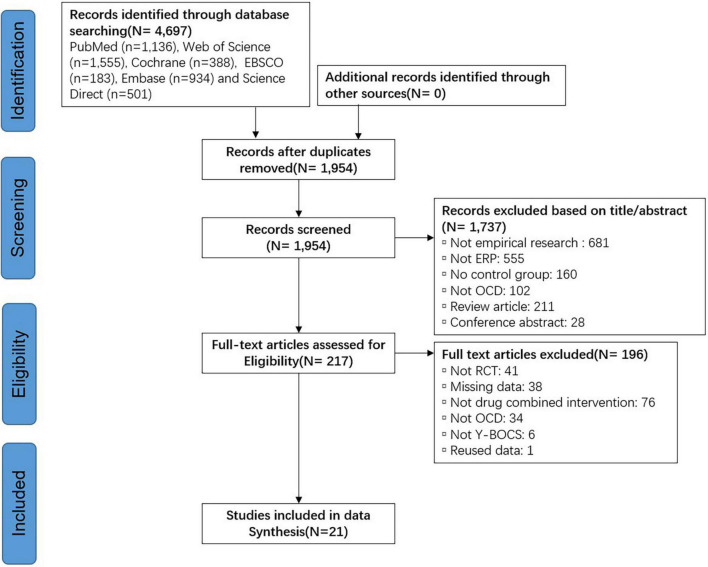
PRISMA flow diagram of article inclusions and exclusions.

The search yielded 4,697 relevant articles, and 21 RCT studies were finally included after duplication, reading of titles and abstracts, and further reading of the full text to eliminate articles that did not meet the inclusion criteria. The article inclusion process and results are shown in Figure 1.

### Risk of bias

**FIGURE 2:**
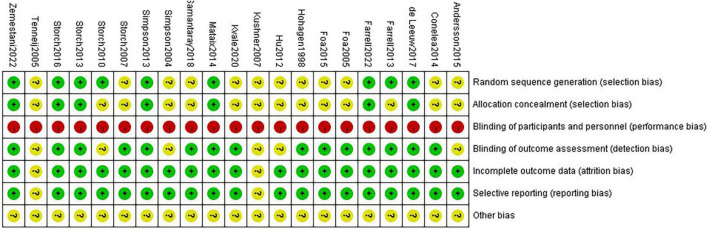
Risk of bias summary.

The included articles were all RCT studies, and there were no significant differences between experimental groups and control groups at baseline. Nineteen studies reported the reasons and numbers of participants who dropped out, and 15 studies were blinded to outcome assessment. Due to the specificity of psychotherapy, a double-blind design was not possible, and the risk of performance bias was high in all studies. Nine studies described the method of random sequence generation, and 7 of them mentioned allocation concealment. Other risks of bias were unclear. The risk of bias report is summarized in Figure 2, “+” means “low bias,” “-” means “high bias,” and “?” means “unclear.”

### Basic characteristics of included articles

The 21 articles included in this study all centered on ERP combined with medication intervention, with 7 studies in children and adolescents and 13 studies in adults. For the control group interventions, nine were pharmacological and twelve were ERP interventions under placebo conditions. Twelve studies reported follow-up data for primary outcome indicators ranging from 1 to 12 months. In addition, 10 studies reported no significant difference between mixed intervention and psychological intervention, 2 studies reported mixed intervention was better than psychological intervention. Six studies reported mixed intervention was better than medication, and 2 studies reported no difference. Basic characteristics of the included studies are shown in Table 1.

**TABLE 1:** Basic characteristics of included studies.

Study	Country	N	Age	Intervention measures		Follow-up time (months)	Ex vs. Con	outcome measure	
							
References		Ex/Con	M (SD)	Ex	Con			OCD	Depression
Andersson et al. (19)	Sweden	64/64	34.6 (12.4)	ICBT + DCS	ICBT + placebo	3	=	Y-BOCS	MADRS-S
Conelea et al. (20)	United States	20/14	13.6 (2.77)	ERP + SSRIs	SSRIs	/	NA	CY-BOCS	/
De Leeuw et al. (21)	Netherlands	19/20	38.1 (14.2)	ERP + DCS	ERP + placebo	/	=	Y-BOCS	/
Farrell et al. (22)	Australia	9/8	13.1 (3.33)	ERP + DCS	ERP + placebo	1,3	>	CY-BOCS	/
Farrell et al. (23)	Australia	49/51	12.3 (2.4)	ERP + DCS	ERP + placebo	1,3,6	=	CY-BOCS	/
Foa et al. (24)	United States	19/27	35.0 (12.2)	ERP + CMI	CMI	/	>	Y-BOCS	/
Foa et al. (25)	United States	30/8	34.5 (13.1)	ERP + SSRIs	RIS + SSRIs	6	>	Y-BOCS	HAM-D
Hohagen et al. (26)	Germany	24/25	37.3 (10.8)	ERP + SSRIs	ERP + placebo	/	>	Y-BOCS	HAM-D
Hu et al. (27)	China	32/35	28.9 (11.2)	ERP + CMI	CMI	3,9	=	Y-BOCS	/
Kushner et al. (28)	United States	14/11	NA	ERP + DCS	ERP + placebo	3	=	Y-BOCS	/
Kvale et al. (29)	Norway	65/31	35.4 (11.4)	ERP + DCS	ERP + placebo	3,12	=	Y-BOCS	PHQ-9
Mataix et al. (30)	United Kingdom	13/14	14.7 (2.1)	ERP + DCS	ERP + placebo	3,6,12	=	CY-BOCS	BDI-Y
Samantaray et al. (31)	India	14/14	25.4 (3.6)	ERP + SSRIs	SSRIs	3,6	=	Y-BOCS	/
Simpson et al. (32)	United States	15/11	34.1 (11.8)	ERP + CMI	CMI	3	>	Y-BOCS	HAM-D
Simpson et al. (33)	United States	37/32	34.3 (12.7)	ERP + SSRIs	RIS + SSRIs	/	>	Y-BOCS	HAM-D
Storch et al. (34)	United States	12/12	32.0 (9.4)	ERP + DCS	ERP + placebo	2	=	Y-BOCS	BDI-II
Storch et al. (35)	United States	15/15	12.2 (2.8)	ERP + DCS	ERP + placebo	/	=	CY-BOCS	CDI
Storch et al. (36)	United States	8/13	11.6 (3.06)	ERP + SSRIs	ERP + placebo	/	=	CY-BOCS	CDRS
Storch et al. (37)	United States	70/72	13.1 (2.93)	ERP + DCS	ERP + placebo	/	=	CY-BOCS	CDRS
Tenneij et al. (38)	Netherlands	34/46	36.7 (12)	ERP + DT	DT	/	>	Y-BOCS	HAM-D
Zemestani et al. (39)	Iran	12/15	36.1 (7.8)	ERP + SSRIs	SSRIs	3	>	Y-BOCS	/

Ex, Experimental; Con, Control; ERP, exposure and response prevention; ICBT, Internet-based cognitive behavioral therapy; DT, drug treatment; SSRIs, selective serotonin reuptake inhibitors; DCS, D-cycloserine; CMI, Clomipramine; RIS, Risperidone; Y-BOCS, The Yale Brown Obsessive Compulsive Scale; CY-BOCS, Children’s Yale-Brown Obsessive-Compulsive Scale; HAM-D, Hamilton Rating Scale for Depression; MADRS-S, Montgomery Åsberg Depression Rating Scale–Self-report; BDI-Y, Beck Depression Inventory for Youth; PHQ-9, Patient Health Questionnaire–9; BDI-II, Beck Depression Inventory — second edition; CDI, Children’s Depression Inventory; CDRS, Children’s Depression Rating Scale; NA, No information.

### Meta-analysis

#### Effect of combination therapy on obsessive-compulsive symptoms in patients with obsessive-compulsive disorder

A total of 1,113 participants were included in the 21 studies that measured the effectiveness of ERP combined with pharmacotherapy on obsessive-compulsive symptoms as post-treatment data. As shown in Table 2, Meta-analysis showed that the heterogeneity *I*^2^-statistic was 86% (*P* < 0.00001), there were significant difference between the combination treatment group and the control group (MD = –3.18, 95% CI: –4.97 to –1.40, *P* = 0.0005).

**TABLE 2:** Treatment efficacy of psychological and pharmacological interventions.

Subgroup name	Studies	Patients	Effect estimate (mean difference [95% CI])	Test for overall effect	Heterogeneity
ERP + DT vs. ERP + placebo	12	698	–0.08 [–1.13, 0.96]	*Z* = 0.16 (*P* = 0.88)	*I*^2^ = 28% (*P* = 0.17)
ERP + DCS vs. ERP + placebo	10	628	0.15 [–0.87, 1.17]	*Z* = 0.29 (*P* = 0.77)	*I*^2^ = 24% (*P* = 0.22)
ERP + SSRIs vs. ERP + placebo	2	70	–2.74 [–6.37, 0.89]	*Z* = 1.48 (*P* = 0.14)	*I*^2^ = 0% (*P* = 0.45)
ERP + DT vs. DT	9	415	–6.60 [–8.35, –4.84]	*Z* = 7.36 (*P* < 0.00001)	*I*^2^ = 58% (*P* = 0.02)
SSRIs: ERP + SSRIs vs. SSRIs	3	89	–7.30 [–9.05, –5.55]	*Z* = 8.17 (*P* < 0.00001)	*I*^2^ = 0% (*P* = 0.38)
RIS: ERP + SSRIs vs. RIS + SSRIs	2	107	–9.72 [–12.21, –7.23]	*Z* = 7.65 (*P* < 0.00001)	*I*^2^ = 0% (*P* = 0.93)
CMI: ERP + CMI vs. CMI	3	139	–4.36 [–7.11, –1.61]	*Z* = 3.11 (*P* = 0.002)	*I*^2^ = 43% (*P* = 0.17)
ERP + DT vs. DT (venlafaxine/paroxetine)	1	80	–5.80 [–9.29, –2.31]	*Z* = 3.26 (*P* = 0.001)	Not applicable
Total	21	1113	–3.18 [–4.97, –1.40]	*Z* = 3.50 (*P* = 0.0005)	*I*^2^ = 86%(*P* < 0.00001)

Publication years, intervention measures, and sample ages were examined as potential moderators of effect size. Meta-regression analyses revealed no significant moderation by publication years (*t* = –1.09, *P* = 0.29) and sample ages (*t* = –0.84, *P* = 0.41). However, intervention measures significantly moderated effect size (*t* = –3.34, *P* = 0.004), indicating that the type of intervention measures was the source of variation.

**FIGURE 3:**
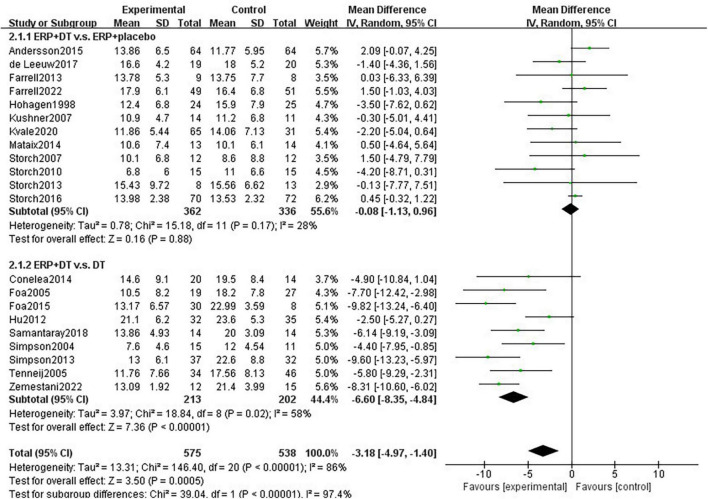
Subgroup analysis of drug type for the effect of combination therapy on the intervention of obsessive-compulsive symptoms in patients with OCD.

For heterogeneity, the studies were classified into 2 subgroups according to the interventions in the control groups. ERP combined with DCS treatment was not significantly different from ERP treatment in the condition of taking placebo (MD = 0.15, 95% CI: –0.87 to 1.17, *P* = 0.77), indicating that DCS drugs do not enhance the effect of ERP intervention in patients with OCD. Given the relatively small number of included studies, it was not sufficient to demonstrate that SSRIs do not enhance the efficacy of ERP, although the difference between ERP combined with SSRIs and placebo was not significant (MD = –2.74, 95% CI: –6.37 to 0.89, *P* = 0.14). ERP combined with drug therapy was significantly better than drug therapy alone, including SSRIs, Clomipramine and Risperidone, with a statistically significant difference (MD = –6.60, 95% CI: –8.35 to –4.84, *P* < 0.00001), see Figure 3.

#### Effect of combination therapy on obsessive-compulsive symptoms in patients with obsessive-compulsive disorder during follow-up

A meta-analysis of 12 studies reporting follow-up data on primary outcome indicators found a total of 588 subjects with *I*^2^-statistic 80% heterogeneity between studies (*P* < 0.00001). The combination treatment group had a greater effect than the control group with a statistically significant difference (MD = –3.17, 95% CI: –5.66 to –0.67, *P* = 0.01).

**FIGURE 4:**
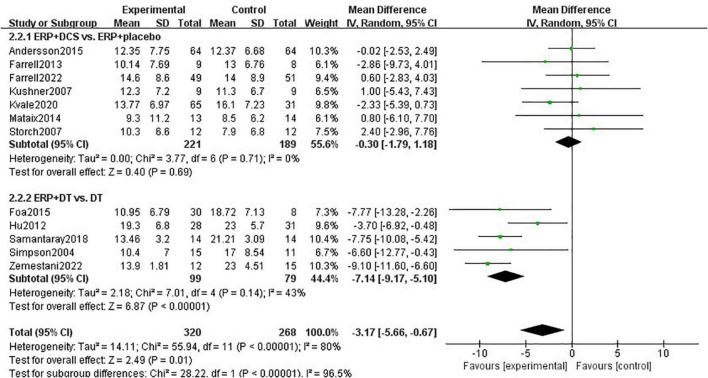
Subgroup analysis of the effect of combination therapy on the intervention of obsessive-compulsive symptoms in patients with OCD during the follow-up period.

For heterogeneity, the studies were divided into two subgroups of DCS and drug treatment, according to the control group interventions. Subgroup analysis showed heterogeneity between studies in the DCS group with *I*^2^-statistic = 0% (*P* = 0.69). There was no significant difference in the long-term efficacy of providing ERP treatment alone versus combined DCS treatment in improving obsessive-compulsive symptoms (MD = –0.30, 95% CI: –1.79 to 1.18, *P* = 0.69). Heterogeneity between studies in the drug treatment group was *I*^2^-statistic = 43% (*P* = 0.14). There is more significant maintenance of treatment effects on the combined treatment than on drug treatment alone during the follow-up period (MD = –7.14, 95% CI: –9.17 to –5.10, *P* < 0.00001). There was heterogeneity between subgroups (*I*^2^-statistic = 96.5%, *P* < 0.00001), indicating that the type of control group was one of the sources of heterogeneity in this subgroup, see Figure 4.

#### Effect of combination therapy on depression in patients with obsessive-compulsive disorder

**FIGURE 5:**
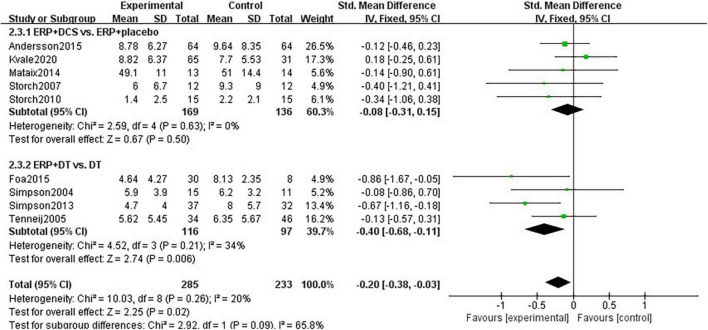
Subgroup analysis of the effect of combination therapy on depression intervention in patients with OCD.

12 studies with a total of 730 subjects were included, with heterogeneity *I*^2^-statistic = 86% (*P* < 0.00001). With regard to heterogeneity, sensitivity analysis found that *I*^2^-statistic decreased to 1% (*P* = 0.44) after excluding one study (37). The difference between the combination treatment group and the control group was significant (SMD = –0.21, 95% CI: –0.38 to –0.04, *P* = 0.02). The studies were divided into 2 subgroups of DCS and drug treatment, according to the control group interventions, and two unclassifiable studies were excluded. Subgroup analysis showed that the heterogeneity between studies in the DCS group was *I*^2^-statistic = 0% (*P* = 0.63). DCS drugs did not enhance the effect of ERP intervention on depression in patients with OCD (SMD = –0.08, 95% CI: –0.31 to 0.15, *P* = 0.50). Heterogeneity between studies in the drug treatment group was *I*^2^-statistic = 34% (*P* = 0.21). ERP combined with drugs improved patients’ depression levels significantly better than providing medication alone (SMD = –0.40, 95% CI: –0.68 to –0.11, *P* = 0.006), see Figure 5.

#### Analysis of article publication bias

**FIGURE 6:**
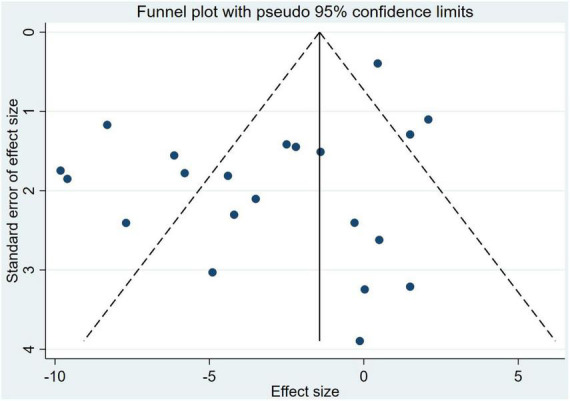
Analysis of article publication bias.

The Egger test indicated that there was a possible publication bias (*t* = –2.52, 95% CI: –4.12 to –0.38, *P* = 0.02) for ERP combined with pharmacological interventions. With regard to the funnel plots, the left-right distribution of the plots was slightly asymmetrical, see Figure 6.

## Discussion

ERP emphasizes layered exposure to fear-inducing stimuli or situations based on patient relaxation, while blocking the patient’s ritualized behaviors and cutting off the reinforcing effects that maintain obsessive-compulsive symptoms, ultimately leading to a gradual fading of the conditioned association between symptoms and the triggering stimulus or situation. ERP treatment is more effective in patients with reduced basolateral amygdala-ventromedial prefrontal cortex functional connectivity in the resting state (40). The amygdala and prefrontal cortex are the underlying structures involved in fear conditioning and regression, and could predict outcomes from ERP. SSRIs are currently the most common drugs used internationally to treat OCD (3). The reduction of obsessive-compulsive symptoms in patients treated with SSRIs medications is associated with reduced metabolic levels in the right caudate nucleus, bilateral orbitofrontal cortex, and bilateral anterior cingulate cortex, and with increased activation in the left thalamus and right insula (41). Nevertheless, if SSRIs medication fails, prompt psychological interventions, such as CBT, behavioral therapy, and ERP, are all acceptable measures, and vice versa (42).

It has been suggested that some patients in studies using ERP-based CBT therapies entered the trial under the condition of continuing their antidepressants, whereas in fact they received the combined treatment (43). The result that CBT’s effect was greater than SSRIs and clomipramine may be attributed to the combined effect of the two treatment modalities (8). Subgroup analysis revealed that ERP combination medication was significantly more effective than providing SSRIs or clomipramine medication alone, with statistically significant changes in Y-BOCS scores before and after treatment, which is consistent with the findings of previous studies (24, 32, 39). In OCD patients with limited benefit from SSRI treatment, combined ERP treatment with risperidone was more beneficial, confirming the findings of prior studies focusing on individuals with refractory OCD (25, 33). Although it appears that a combination of pharmacological and psychological treatment is effective, there is no convincing evidence that ERP alone is more effective than combination treatment. A systematic review comparing behavioral and pharmacological therapies similarly stated that combination therapy had little benefit over behavioral therapy in an outpatient setting, but that it was at least superior to pharmacological therapy (12).

Regarding the long-lasting mechanism of treatment effects, one study reported no significant difference between drug and combination therapy groups (25), while others found that combination therapy was significantly better than drug therapy (31, 39). The results of Subgroup analysis revealed that the majority of OCD patients maintained modest efficacy during the follow-up period after ERP combination medication therapy. The follow-up duration included in this study was, however, relatively short, and some of the articles did not offer statistics on long-term treatment or relapse prevention. Since the majority of OCD patients are chronic, more follow-up statistics on long-term treatment are required to determine the duration of symptom improvement without relapse following combination therapy.

Hitherto, the results of clinical studies of DCS enhanced ERP for OCD have been inconsistent. Farrell et al. reported significant improvement in OCD symptoms with DCS enhanced ERP treatment compared to placebo (22). However, Subgroup analysis revealed that DCS does not have a mechanism for enhancing ERP efficacy and did not significantly differ from the placebo group, which is in line with prior findings (19, 21, 29). Nevertheless, it has been suggested that antidepressants may block the effect of DCS in promoting fear extinction, with a higher percentage of symptom improvement in the DCS group among patients not taking antidepressants, and DCS may be utilized as an intensive CBT treatment approach in patients with OCD who are not on antidepressants (19). The analysis revealed no significant difference in the reduction of Y-BOCS scores between the DCS enhanced and placebo enhanced ERP groups during the follow-up period, requiring further confirmation of the mechanism underlying the long-lasting effects of DCS enhanced ERP treatment. However, it has been argued that the augmentation impact of DCS is time-limited, and that the most significant benefits may be an earlier gain in ERP treatment effect, a reduction in the number of treatments required, lower treatment costs and dropout rates (44).

Due to the presence of compulsions and counter-compulsions, patients with OCD commonly suffer anxiety. Furthermore, for ERP treatment to be effective, it is a precondition that patient actively confronts the stimulus or situation (45), which may aggravate the patient’s anxiety during ERP treatment. Compulsion is associated with a large number of psychiatric comorbidities, with anxiety and mood disorders being more common (46). Therefore, patients with comorbidities were not excluded from this study, and several included papers reported co-morbid anxiety or depression in some patients (19, 21, 22, 30, 33–35, 39). The supramarginal gyrus is thought to be related to emotion processing, and its dysfunction may affect patients’ sensitivity to uncomfortable emotions or anxiety, as well as be responsible for exacerbating the co-morbidity of obsessive-compulsive and anxious emotions (47). A Meta-analysis found that neither ERPs nor SSRIs significantly reduced anxiety and depressive symptoms (48). In contrast, Meta-analysis revealed that ERP combined with pharmacotherapy significantly improved depressive symptoms in patients. Alleviating obsessive-compulsive symptoms can ameliorate depressive symptoms to a great extent (49). Approximately one-third of OCD patients currently have comorbid major depressive disorder and roughly two-thirds suffer from a lifetime comorbidity (16). When compulsions are accompanied by a depressive mood, it can interfere with the completion of homework assignments and the development of therapeutic habits during ERP treatment. Combining antidepressants and psychotherapy for OCD patients who have significant depressive symptoms may be more effective than monotherapy (50). In particular, for patients with severe obsessive-compulsive co-morbid depression who do not respond well to therapy alone, a combination of CBT involving ERP and SSRIs is frequently recommended (8).

Recent research evaluated the efficacy and tolerability of three commonly used atypical antipsychotics (risperidone, paliperidone, and aripiprazole) as reinforcing treatments for OCD, which are more likely to be used clinically as augmentation agents to treat patients when SSRIs and ERP have limited treatment effect (51). In addition, ketamine plays an important role in the cortico-striato-thalamo-cortical loop as a potent N-methyl-D-aspartic acid receptor antagonist and glutamatergic modulator. Given the limitations of current OCD treatment and the new evidence that glutamate may improve depression and play a role in OCD pathophysiology, ketamine has emerged as an alternative drug in this field (52).

Exposure to intrusive thoughts poses some risk, and high levels of emotional arousal and discomfort may account for the high ERP dropouts. In contrast to traditional therapies that attempt to eliminate distress directly, acceptance and commitment therapy (ACT) focuses on increasing psychological flexibility, comprehending anxiety, and altering how distress is dealt with. Patients treated with ACT and combination therapy showed more significant improvements in post-treatment obsessive-compulsive symptoms and experiential avoidance compared to those treated with SSRIs alone, according to an RCT (53). The small number of studies, however, does not support a more significant intervention impact of ACT combined with SSRIs. Future research could focus on applying ACT to various subtypes of OCD in order to further validate the effectiveness of ACT in conjunction with medication for OCD patients.

## Conclusion

This study confirmed the feasibility of ERP combined with medication in improving obsessive-compulsive symptoms and depressive mood in patients with OCD, but was insufficient to demonstrate that DCS enhances the efficacy of ERP. The inclusion of data from 10 countries worldwide further enhances the generalizability of the results, but there are some limitations. First, the relatively low number of studies and sample sizes inevitably led to lower detection power. Secondly, children and adolescents were included in the analysis along with adult patients. Finally, some studies were excluded for data could not be extracted, which affected the analysis results to some extent. Future studies with larger sample sizes should be designed to validate the findings of this study.

## Data availability statement

The raw data supporting the conclusions of this article will be made available by the authors, without undue reservation.

## Author contributions

LM: manuscript writing, study search, data collection, and data analysis. MH: study search, data collection, and data analysis. LL, YW, ZL, and JZ: selected the studies, extracted the data, and assessed the risk of bias. All authors contributed to the article and approved the submitted version.
